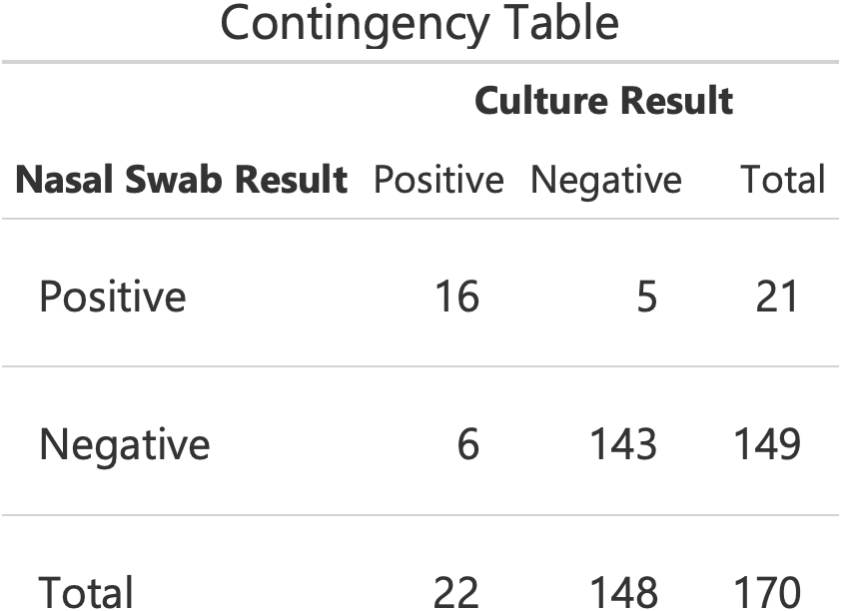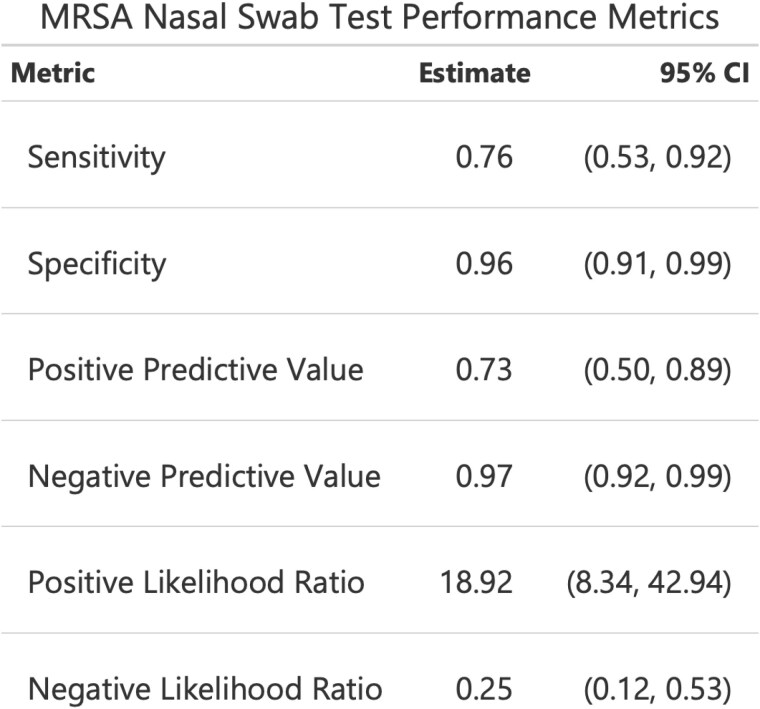# 31 Reliability of Methicillin-Resistant Staphylococcus aureus (MRSA) Nasal Swab Screening for Predicting MRSA Burn Cellulitis

**DOI:** 10.1093/jbcr/iraf019.031

**Published:** 2025-04-01

**Authors:** Max Silverstein, Yvonne Karanas, Clifford Sheckter

**Affiliations:** Division of Plastic and Reconstructive Surgery, Stanford University; Santa Clara Valley Medical Center; Santa Clara Valley Medical Center

## Abstract

**Introduction:**

Infection is the primary cause for death after 24 hours in patients admitted to burn centers, with cellulitis trailing only pneumonia as the most common source of sepsis. Staphylococcus aureus is the organism isolated most frequently from cellulitic burns. Over decades, methicillin-resistant Staphylococcus aureus (MRSA) has become endemic in burn units, resulting in the frequent initiation of empiric vancomycin therapy. MRSA polymerase chain reaction (PCR) nasal swab screening rapidly identifies patients who are MRSA-colonized, with the goal of predicting the presence of MRSA in any future infections. While nasal swab screening is standard-of-care for de-escalating MRSA coverage in cases of pneumonia, its predictive value has not been studied in the burn context. We hypothesized that MRSA nasal swab screening would have high negative predictive value for ruling-out the presence of MRSA in cultures from infected burns.

**Methods:**

We performed a retrospective review of all patients admitted to a verified burn center who were treated with antibiotics for a cutaneous burn infection or undifferentiated sepsis between 2021 and 2022. Our institutional policy requires nasal MRSA swab screening of all patients on admission for purposes of rooming and contact precaution. All patients who developed a burn-related wound infection and were tested via wound culture prior to the initiation of antibiotics were included. The diagnostic characteristics of nasal swab screening for predicting the presence of MRSA on wound culture were calculated. Chi-square testing and odds ratios determined statistical significance at p< 0.05.

**Results:**

170 patients underwent MRSA nasal swab screening and were treated for burn-related infection during the study period. Median age was 53 years, median TBSA was 6.0%, 70% were men, and 77% underwent surgical treatment. MRSA was detected on 12% of nasal swabs and in 12% of wound cultures. PCR nasal swabs had a sensitivity of 76%, specificity of 96%, positive predictive value of 73%, and negative predictive value of 97% for the presence of MRSA on wound culture. At the time of wound culture, a positive MRSA nasal swab was associated with increased likelihood of MRSA infection with an odds ratio of 76.3 (95% CI 20.9-278.3, p< 0.001).

**Conclusions:**

PCR nasal swab screening had a high negative predictive value for ruling-out the presence of MRSA in infected burns. A negative MRSA nasal swab on admission is a reliable indicator to de-escalate MRSA coverage when treating patients with infected burns, even prior to confirmation via bacterial wound culture.

**Applicability of Research to Practice:**

Burn centers should employ universal MRSA nasal swab screening and de-escalate MRSA antibiotic coverage when treating burn infections in patients with a negative nasal swab. This has potential to improve antibiotic stewardship and reduce vancomycin-related complications including acute kidney injury.

**Funding for the Study:**

The senior author is is supported by a grant from the National Institutes of Health. No specific funding was received for this study.